# Reintroduction of Clozapine following Neuroleptic Malignant Syndrome in a Young Patient with Resistant Schizophrenia

**DOI:** 10.1155/2024/9936663

**Published:** 2024-05-10

**Authors:** Miriam Chandad, Rajae Chlihfane, Safae Kodad, Bouchra Oneib, Fatima Elghazouani

**Affiliations:** Department of Psychiatry, Faculty of Medicine and Pharmacy, Mother and Child Health and Mental Health Research Laboratory, Mohammed I University, Oujda, Morocco

## Abstract

The incidence of neuroleptic malignant syndrome justifies the immediate discontinuation of the drug in question and the reinstitution of therapy with another drug. In the case of resistant schizophrenia treated with clozapine, there are insufficient therapeutic options. We report the case of a young patient followed up for resistant schizophrenia who developed neuroleptic malignant syndrome after 5 years of therapy with clozapine. Clozapine therapy was successfully reinitiated, and the dosage was increased to 300 mg/day over 62 days. In light of this clinical case and a review of the literature, we report the possibility of reintroducing clozapine following an incidence of malignant syndrome in patients with resistant schizophrenia with respect to certain rules; in particular, a slow increase in dose after a reasonable period of time and close monitoring.

## 1. Introduction

Clozapine is a second-generation antipsychotic drug with major indications for treatment-resistant schizophrenia. Furthermore, clozapine has demonstrated additional benefits other than its antipsychotic effects. It reduces suicidal tendencies in patients with schizophrenia and alleviates tardive dyskinesia [1, 2].

However, patients and clinicians hesitate to initiate this medication because fear of serious or even fatal adverse effects, such as neuroleptic malignant syndrome (NMS), which requires rigorous monitoring.

NMS typically manifests within the 2 weeks of commencing antipsychotic treatment or following dosage adjustment. Its characteristic presentation involves muscular rigidity, elevated body temperature, impaired consciousness, and autonomic dysfunction as observed during clinical examinations. Laboratory tests often reveal elevated levels of leukocytes and creatine phosphokinase (CPK) levels. This severe and unpredictable reaction that constituted a medical emergency.

Guidelines recommend immediate discontinuation of the causative molecule and prohibition of its resumption, owing to the risk of recurrence of this deadly effect.

When confronted with this predicament, clinicians find themselves in a difficult position, weighing the potential benefits of the molecule against the associated risks.

We report the successful reinitiation of clozapine treatment in a young patient with treatment-resistant schizophrenia following an NMS episode. Our objective was to provide encouragement and guidance to clinicians facing similar challenges in clinical practice.

## 2. Case Presentation

We report the case of a 35-year-old unmarried man who was followed-up for resistant schizophrenia. The patient had no significant personal, family, medical, or surgical history. Other than tobacco, he did not consume any psychoactive substances. The symptoms of schizophrenia initially appeared when he was 23 years old and were characterized by gradual onset of insomnia, neglect of personal hygiene, incoherent speech, mystic-religious delusions, paranoid ideation, and a tendency toward verbal and later physical aggression. The patient was initially administered oral haloperidol (12 mg/day) and chlorpromazine (200 mg/day). This treatment regimen yielded favorable improvement in the positive symptoms of schizophrenia; however, it did not result in satisfactory socioprofessional integration. Unfortunately, owing to poor medication adherence, the patient experienced multiple relapses over time, which progressively worsened in severity and showed resistance to the prescribed antipsychotics.

The diagnosis of resistant schizophrenia was retained after 5 years of evolution. Clozapine was administered to the patient. At a dosage of 350 mg/day, a favorable improvement in positive symptoms was noted, without resuming professional activity for 3 years. Following a stock shortage at the pharmacy, the patient stopped treatment for 6 months, which was followed by a gradual relapse. As soon as the drug became available, his mother administered 200 mg/day of clozapine without medical advice. After a few days, the patient was brought back to the psychiatric emergency room, bedridden and unconscious with impossible contact. General examination revealed that the patient was afebrile, with signs of dehydration and muscle rigidity.

The blood test showed an inflammatory syndrome (C-reactive protein (CRP) 30.36 mg/L (normal values (NV): 0–5 mg/L) and neutrophil hyperleukocytosis 9,410/uL (NV: 1,500–7,000/uL)), rhabdomyolysis (CPK 7,395 UI/L (NV: 30–200 UI/L), lactate dehydrogenase (LDH) 512 UI/L (NV: 125–243 UI/L)), and a twofold increase in aspartate aminotransferase (AST). Blood ionogram, renal assessment, cyto-bacteriological examination of urine (CBEU), and the computerized tomography were performed, and the results were not remarkable. No infectious or metabolic etiology was identified and the diagnosis of NMS was retained. The patient was admitted to the medical intensive care unit. The clinical evolution was marked by the appearance of a generalized tonic–clonic convulsive seizure explained by the occurrence of hyponatremia at 127 mEq/L (NV:136–145 mEq/L) and hypokalemia at 3.1 mEq/L (NV: 3,5–5,1 mEq/L), which was resolved.

**Figure 1:**
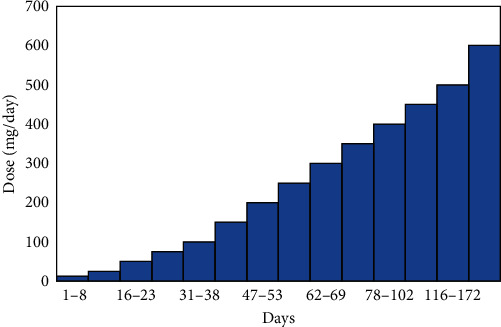
The patient's clozapine restart strategy.

Following clinical and biological stabilization (CPK decreased by 50% after 72 hr and was normalized after 15 days), the patient was transferred to the psychiatric ward; where he presented with disorganization and instability. Despite the gradual introduction of olanzapine as an alternative treatment, it failed at a dose of 20 mg/day. Electroconvulsive therapy (ECT) has been proposed as an alternative; however, consent has not yet been obtained. Given this therapeutic impasse, strict and rigorous clinical and biological titration and monitoring (Figure 1) were implemented during the reintroduction of clozapine. The clinical picture improved with a dose of 600 mg/day, and psychoeducation of the family was recommended to prevent future incidents.

## 3. Discussion

The present case demonstrates the feasibility of reintroducing clozapine following an incidence of NMS in patients with resistant schizophrenia, with limited therapeutic choices. This highlights the importance of clinicians carefully weighing the risks and benefits of rapid clozapine titration.

Clozapine is the treatment of choice for patients resistant to schizophrenia. Clinical guidelines recommend progressive titration to reduce the risk of adverse effects, such as hypotension, convulsions, agranulocytosis, NMS, and myocarditis [3]; however, this may delay the effective control of severe psychotic symptoms.

In our clinical case, clozapine 200 mg/day induced NMS, whereas subsequent progressive titration prevented the recurrence of this serious adverse effect. Rapid titration of clozapine has implications for reducing the costs associated with prolonged hospitalization; and facilitating the rapid control of psychotic symptoms, thus avoiding the risky use of multiple psychotropic drugs. Nevertheless, it is important to note that data supporting the practice of rapid clozapine titration are very limited [4, 5], prompting clinicians to carefully weigh up the associated risks and benefits.

Based on our observations of various cases reported in the literature [6–8], a longer delay between the start of treatment and NMS onset may be a risk factor for the recurrence of this syndrome, although this needs to be confirmed in further studies.

In our case study, NMS occurred after 5 years of clozapine therapy; in a patient with the following risk factors: male sex and self-administration of 200 mg/day clozapine without titration. Dehydration on clinical examination cannot be considered a precipitating factor because, chronologically, it was not clear whether it preceded NMS onset or was a consequence.

In the literature, there are no studies on the reintroduction of clozapine in patients who developed NMS, apart from a few rarely reported cases (Table 1) in which the outcome was always excellent. However, in the literature, not enough cases have failed, either because they do not exist or that only excellent results have been reported. Therefore, the resumption of treatment remains controversial. Nonetheless, considering the limited therapeutic options available for treatment-resistant patients, and the unavailability and refusal of ECT, clozapine therapy can be reattempted with due consideration for the following set of rules:

1. Restarting of clozapine following an NMS incidence should be deferred.
2. Concomitant therapy with neuroleptics and lithium or anticholinergic drugs, or paroxetine, should be avoided [14, 15].
3. Dose increase must be gradually progressive to prevent NMS recurrence.
4. Clinical and biological monitoring, including monitoring of CPK levels, must be performed very close to the early detection of any signs of NMS.
5. Patient counseling on the importance of good hydration is necessary to prevent dehydration, which can trigger NMS.

## 4. Conclusion

This case illustrates the successful restart of clozapine treatment following NMS with adherence to specific measures, including providing appropriate education to patients and their families regarding medication safety and adherence.

## Figures and Tables

**Table 1:** List of cases in the literature where clozapine was restarted after neuroleptic malignant syndrome.

Case report	Number of patients	Age/sex	Resumption strategy	Reintroduction interval (weeks)
Huang [8]	1	34/F	2-week titration to 250 mg	2
Tsai et al. [9]	1	35/F	Gradual dose increase	1
Anderson and Powers [10]	1	26/F	Not mentioned	2
Goates and Escobar [11]	1	29/M	2–3 weeks titration from 25 to 500 mg	1, 5
Chatterton et al. [12]	1	49/M	Not mentioned	36
Anbalagan et al. [13]	1	24/F	2-week gradual titration	2

## Abbreviations

NMS: Neuroleptic malignant syndrome
ECT: Electroconvulsive therapy
CPK: Creatine phosphokinase
CRP: C-reactive protein
LDH: Lactate dehydrogenase
AST: Aspartate aminotransferase
CBEU: Cyto-bacteriological examination of urine.
